# Using the Simulated Patient Method to Evaluate the Community Pharmacy Management of Childhood Diarrhoea: A Systematic Review

**DOI:** 10.21315/mjms2023.30.5.5

**Published:** 2023-10-30

**Authors:** Hananditia Rachma Pramestutie, Susi Ari Kristina, Lutfan Lazuardi, Anna Wahyuni Widayanti

**Affiliations:** 1 Doctoral Program in Pharmacy, Faculty of Pharmacy, Universitas Gadjah Mada, Yogyakarta, Indonesia; 2 Department of Community Pharmacy, Faculty of Medicine, Universitas Brawijaya, Malang, Indonesia; 3 Department of Pharmaceutics, Faculty of Pharmacy, Universitas Gadjah Mada, Yogyakarta, Indonesia; 4 Department of Health Policy and Management, Faculty of Medicine, Public Health and Nursing, Universitas Gadjah Mada, Yogyakarta, Indonesia

**Keywords:** simulated patient, community pharmacist, diarrhoea, children

## Abstract

The simulated patient method has been widely used to assess community pharmacy practice in the management of childhood diarrhoea. In such a process, a community pharmacist is required to explore a patient’s history, choose the right medication and provide drug-related information. The aim of this review was to evaluate the aforementioned practice. A comprehensive literature search was carried out over Sage Journal, PubMed, ScienceDirect and Google Scholar, and the analysis was conducted in accordance with the Preferred Reporting Items for Systematic Reviews and Meta-Analyses. Eligible articles were those published from 2011 to 2021 and original studies that used the simulated patient method to examine the pharmaceutical services provided by pharmacists in relation to childhood diarrhoea. The eight studies that satisfied the eligibility criteria were reviewed. These investigations were undertaken in Brazil, Nigeria, Turkey, Ethiopia and Pakistan. Five of the studies focused on history taking with regard to the characteristics of diarrhoea and revealed that the evaluated pharmacists asked about patient histories. In terms of therapy, three studies indicated that the evaluated pharmacists recommended the administration of oral rehydration salts. Pharmacies should improve their history-taking process, provide drug-related information and recommend therapies to increase the knowledge of simulated patients about diarrhoea treatment in children.

## Introduction

Diarrhoea can be defined as a condition in which the faeces passed by patients are of a soft or liquid consistency; diarrhoeal stool can be fully composed of water and defaecation happens frequently (usually three or more times) within a day. Diarrhoea causes other changes in bowel movement (1, 2). It is one of the leading causes of death all over the world, resulting in 5 million–10 million fatalities per year. The highest prevalence of diarrhoea occurs in children aged 12 months old–23 months old, followed by children aged 6 months old–11 months old and 23 months old–45 months old. Diarrhoea is contracted mostly by children aged 6 months old–35 months old because this is the period at which they start playing actively and are exposed to the risk of infection (3, 4).

Diarrhoea can be classified into acute and chronic diarrhoea, each requiring different ways of handling and treatment. The improper handling and treatment of the condition can be a serious problem, and not all parents understand first aid for childhood diarrhoea. These observations are supported by a study in Tanzania regarding the knowledge of mothers and caregivers regarding diarrhoea management in 5-year-old children. Many of the respondent mothers or caregivers (71%) believe in the use of traditional medicine, namely, guava leaves and fruit, to treat the condition. With regard to chemical medicine, nearly half of the respondents (48.9%) choose metronidazole as diarrhoeal therapy (5). Many parents self-medicate children with diarrhoea by buying medicine from pharmacies, but they initiate this process by consulting a pharmacist, as shown in several studies (6, 7). Pharmacists therefore play an important role in providing appropriate therapeutic recommendations and drug-related information for paediatric patients with diarrhoea. The administration of therapy is adjusted to a patient’s clinical condition.

The patient simulation method can be used to determine the effectiveness of pharmacists in providing drug-related information and therapeutic recommendations for children with diarrhoea (8). This method serves as a solution because pharmacists do not realise that they are being evaluated, thereby enabling the acquisition of genuine results. It is hoped that with this method, evaluators can ascertain how pharmacists actually deliver pharmaceutical services related to the management of diarrhoea in children and select treatments according to scenarios designed by researchers (9).

No study has been carried out on the evaluation of pharmacists’ provision of drug-related information on diarrhoea in children, wherein simulated patients act as parents of afflicted children, with guidance from a scenario prepared by a researcher. The closest works in this respect recruited simulated patients for assessment purposes, but they did not explore all the components of drug information extraction (10, 11). Pharmacists should acquire complete information so that they correctly offer recommendations for treating diarrhoea in children (12). Many respondents have recommended the administration of antibiotics to treat diarrhoea in children and others suggested therapies such as antimotility medication, probiotics and oral rehydration salts. Several respondents have also suggested the use of drugs that have been removed from their original packaging (13).

A systematic review of studies that used the patient simulation method can be conducted to determine how pharmacists provide drug-related information and therapeutic recommendations for children with diarrhoea. However, no such review has been carried out. To address this deficiency, the present research was conducted to systematically evaluate simulated patient based studies on paediatric diarrhoea, with focus directed specifically to the history taking process, the provision of drug-related information and the therapies recommended by pharmacists.

## Methods

### Search Strategies

Articles published in the last 10 years (2011–2021) were searched over the databases of Sage Journal, PubMed, ScienceDirect and Google Scholar. Given the large scale of these databases, broad, thorough and inclusive terms were used in the search. Specifically, the keywords employed were ‘simulated patient’ OR ‘standardised patient’ AND ‘community pharmacists’ AND ‘diarrhoea’ AND ‘childhood’ OR ‘children’. Mendeley was used to eliminate duplicate citations, after which the generated articles were organised.

### Study Selection

The Preferred Reporting Items for Systematic Reviews and Meta-Analyses were used as guidance in conducting and presenting the systematic review. The inclusion criteria were articles published in English, randomised controlled trials, cross-sectional studies, cohort studies, papers discussing history taking by pharmacy professionals for paediatric diarrhoea patients and articles discussing information given by pharmacy professionals in connection to children with diarrhoea. This theme discusses drugs recommended for paediatric diarrhoea. The exclusion criteria were articles for which the full texts are unavailable, literature reviews, handbooks, book chapters and reports. The articles yielded by the search were then grouped and analysed. Two research team members (HRP and SAK) independently screened full-text papers for inclusion, and LL and AWW examined differences in screening. At each level of the screening process, the reasons driving the rejection and documentation of articles were specified.

### Data Extraction and Synthesis

Standard data extraction forms were used to document the authors of the sampled studies, years of publication, countries where the studies were carried out and study designs. The forms also contained brief explanations of the techniques, primary outcome measures and conclusions used and drawn by the researchers. The information was compiled in a narrative format, and the findings of each study were examined in terms of three dimensions: patient history taking by pharmacists, information provision by pharmacy professionals and drug recommendations.

### Methodological Quality Assessment

The Checklist for Reporting research using Simulated Patient (CRiSP) methodology was used to assess the methodological quality of the papers. The CRiSP can be used as a reporting guideline for authors using the simulated patient methodology. The checklist consists of 23 questions with five possible responses: ‘yes’, ‘no’, ‘unclear’, ‘not applicable’ and ‘partially complete’; a space for brief remarks is also provided (14). Two researchers independently assessed the quality of a given study. Disagreements among team members (HRP, SAK, LL and AWW) were handled through dialogue.

## Results

**Figure 1:**
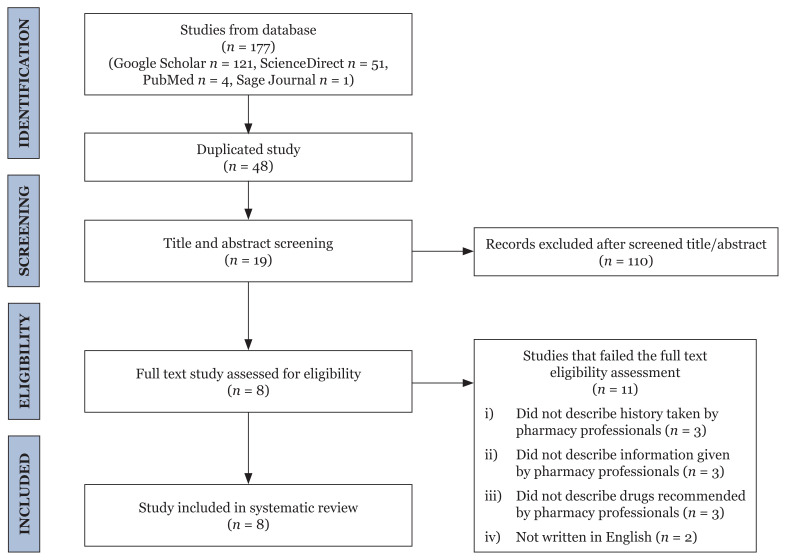
Prisma diagram

A total of 177 distinct studies were generated from the search (121 from Google Scholar, 51 from ScienceDirect, four from PubMed and one from Sage Journal). Forty-eight studies had the same title and were therefore excluded. After the evaluation of titles and abstracts, 19 were deemed compliant with the requirements for inclusion. Following a thorough analysis, eight studies were retained for the systematic review (Figure 1).

### Study Characteristics

The eight studies were undertaken in Brazil (15), Nigeria (11), Turkey (16), Ethiopia (17–20) and Pakistan (21). All the explorations were conducted in community pharmacies and all were of a cross-sectional design, developed specifically to examine the counselling behaviours of pharmacists and their staff when confronted with diverse scenarios involving the treatment of diarrhoea in children (Table 1).

### Methods for Assessing Community Pharmacy Practice

The studies used various research approaches, among which three were intended to measure the quality of counselling provided by pharmacists and pharmacy staff. Specifically, two studies used the patient simulation method and provided simulated patients with a checklist of items related to the counselling provided by pharmacists/pharmacy staff (16, 21); another two employed the simulated patient method and conducted in-depth interviews with simulated patients after they visited pharmacies (18, 19); and four used a combination of patient simulation and questionnaire administration after simulated patient visits to pharmacies (11, 15, 17, 20).

To determine interactions between simulated patients and pharmacists/pharmacy staff regarding history taking, the provision of drug-related information and recommended therapies, the researchers used data collection sheets, audio recorders and audiovisual recorders. Five studies used data collection sheets, which were filled in following simulated patient visits to the pharmacies where the investigations were conducted (11, 16, 17, 20). Two studies carried out audio recording during simulated patient visits to pharmacies as consultations regarding the treatment of children’s diarrhoea took place (18, 19). In one study, the researcher operated an audiovisual recording device (hidden micro camera) while the simulated patient interacted with pharmacists/pharmacy staff (15). The researchers and simulated patients documented the results on data collection sheets (Table 1).

### Scenarios

All the simulated patients visited pharmacies guided by researcher-prepared scenarios, which all featured the management of childhood diarrhoea. They visited pharmacies asking for diarrhoea medicine to give to toddlers, but only one study involved a simulated patient who asked about the treatment of diarrhoea in infants aged 7 months (16). The simulated patients visited pharmacies, asked to see the pharmacists working in the establishments and then consulted them about treating diarrhoea in children. In cases wherein no pharmacist could meet with a simulated patient, the latter made their treatment concerns known to pharmacy staff. The simulated patients explained the symptoms experienced by their children to the pharmacists/pharmacy staff. Only one simulated patient stated that their child had both fever and diarrhoea (15). All the simulated patients visited pharmacies only once. Concerning awareness of visits made by the simulated patients, six studies did not notify the pharmacists of simulated patient arrivals (covert) (11, 17–21), whereas two distributed consent forms to the pharmacists before visits by the simulated patients (consented) (15, 16) (Table 1).

The simulated patients were trained in different ways across the studies. These individuals were undergraduate pharmacy students (*n* = 3), a pharmacy graduate (*n* = 1), a young mother and caregiver (*n* = 1), a clinical pharmacist (*n* = 1) and a non-healthcare worker (*n* = 1).

### Counselling for Simulated Patients

A pharmacist or pharmacy staff should enquire about patient history, explain drug-related information and give therapeutic recommendations at each stage of the counselling process. History taking by pharmacists and pharmacy staff varied across the studies. The pharmacists or pharmacy staff enquired into the characteristics of diarrhoea experienced by children, that is, the frequency of bowel movement (11, 15–17), the presence of mucus and blood in the stool (11, 16, 17, 20) and the duration of a patient’s diarrhoea (15–17, 20). In five studies, the pharmacists/pharmacy staff obtained the medication histories of the simulated patients (15, 16, 18, 19, 21), whereas in four studies, such inquiries were restricted to the patients’ ages (11, 16, 17, 20). Some pharmacists/pharmacy staff asked about patient symptoms, such as fever (11, 16, 17, 20) and vomiting (16, 20).

The pharmacists provided various types of information. In most of the studies, the pharmacists explained information about pharmacological therapy, such as dosage (15, 17–20), drug administration frequency (15, 17–20), indications (15, 18, 19) and adverse drug reactions (17–19). In two studies, the pharmacists offered non-pharmacological advice (18, 19), and in one research, the pharmacist instructed the patients on how to store drugs (20). The pharmacists also provided different therapeutic recommendations. In three studies, the pharmacists suggested the administration of oral rehydration salts (11, 15, 16), and in another three investigations, the pharmacists recommended a combination of oral rehydration salts and zinc (17–19). Loperamide, anthelmintics and metronidazole were recommended by pharmacists in each of three studies (15, 17–19, 22) (Table 2).

### Article Quality

Article quality, which was evaluated using the CRiSP, varied across the studies. None of the explorations fulfilled all the CRiSP requirements, and only four described the study designs used (15, 17, 20, 22). Two studies did not report the number of simulated patients recruited (17, 21) but all of them provided details about the featured scenarios, including patient characteristics; patient prompts, scripts and props; models for the delivery of simulated patient assessment; and ethical approval processes, consent from participants and ways of maintaining confidentiality (Table 3).

## Discussion

The use of the simulated patient method in eight studies on medication for diarrhoea in children in the community pharmacy setting was systematically examined in this review. Specifically, it probed into pharmaceutical practices in different countries in terms of history taking, therapeutic recommendation and drug-related information provision by pharmacists. The simulated patient method is a practical and straightforward approach to assessing the counselling practices of pharmacists and pharmacy staff in relation to the treatment of childhood diarrhoea, with simulated patients visiting community pharmacies guided by scenarios designed by researchers. By directly visiting pharmacies, simulated patients can obtain information on counselling in connection to medication history, drug-related information and drug recommendations for paediatric diarrhoea. In community pharmacies, pharmacists provide recommendations for treating minor illnesses because patients first meet with a pharmacist before consulting a doctor. Diarrhoea in children is an example of a mild disease for which more people go to pharmacies for suggestions regarding therapy (23).

Six studies employed the simulated patient method to covertly evaluate pharmacist and staff practice behaviours. The findings revealed that the primary objective underlying the use of the simulated patient approach was assessment, rather than instruction meant to enhance the practical skills of pharmacists and their staff. This result also underlines the issue that despite the use of simulated patients in evaluating pharmacy practice, little research has been paid to performance feedback and training/pharmacy education as a means of moulding pharmacists and their employees (24). The most prevalent data collection technique in the reviewed papers was note-taking or checklist completion, and data from these were documented as quickly as possible following simulated patient sessions. Generally, self-completed questionnaires are the most popular data-gathering tools in research on pharmacy practice. However, because of the fallibility of recall and recollection, difficulties can develop from the interval between observation and data recording (9). Two studies made voice recordings of consultations regarding the treatment of children’s diarrhoea during simulated patient visits to pharmacies, and one used an audiovisual recording device (hidden micro camera). Instead of depending only on a simulated patient or researcher, a study used audio recording to reduce selectivity and inferences related to researcher observation and recording, thus improving the understanding of specific occurrences during simulated patient encounters (25).

The overall results indicated that most of the pharmacists and pharmacy staff exercised poor pharmaceutical practices in terms of obtaining patient history, offering medication recommendations and providing drug information to simulated patients who acted as parents of children with diarrhoea. Pharmacists must know the different types of diarrhoea, their symptoms and therapeutic management for children to be able to offer appropriate suggestions. To ensure the accuracy of therapeutic recommendations, pharmacists should acquire a complete patient history (3), including details such as disease onset, duration, severity, frequency and the presence of other symptoms, such as fever and vomiting and stool consistency and composition (e.g. bloody or watery stool). It is also necessary to enquire about a patient’s identity (name, age and weight) and whether they have taken certain drugs and have drug allergies (26).

The findings reflected that in most of the studies, the pharmacists did not obtain a complete patient history. They enquired into the characteristics of diarrhoea experienced by children, namely, the frequency of bowel movement (range = 8%–44.2%), the presence of mucus and blood in the stool (range = 1%–49.6%) and the duration of patient diarrhoea (range = 18%–67.3%). Pharmacists need to determine the duration of diarrhoea in children because there are two types of the disease: acute and chronic diarrhoea. If a patient has diarrhoea for less than 14 days, the condition would be diagnosed as acute diarrhoea. The chronic form lasts for more than 14 days. Acute and chronic diarrhoea require different ways of handling and treatment (27). The results are inversely proportional to research conducted in Germany, where 48.7% of the evaluated pharmacists obtained information on diarrhoea duration (28). A study conducted in Iraq found that 80% of the assessed pharmacists asked about diarrhoea in simulated patients who visited pharmacies (26).

Medication history is one of the essential issues that pharmacists must discuss with patients. Obtaining this information is aimed at ruling out drugs that can cause diarrhoea, avoiding the duplication of therapy and preventing drug interactions (23, 29). As indicated by the results, in five studies, the pharmacists acquired information related to patients’ medication histories (range = 1%–32.5%). The results are almost similar to research conducted in Germany, wherein as many as 32.7% of the pharmacists derived histories related to patient treatment (28).

Diarrhoea is accompanied by several symptoms, including nausea and fever. Nausea often co-occurs in cases of cholera caused by bacterial toxins, while diarrhoeal fever in children can stem from infection by certain bacteria (30). The results showed that in four studies, the pharmacists determined whether the afflicted children had a fever (range = 2%–41.6%) and whether they experienced nausea and vomiting (range = 5%–19%). Pharmacists should look into the symptoms that accompany diarrhoea to provide both pharmacological and non-pharmacological therapeutic recommendations for alleviating these symptoms (23).

The management of mild to severe diarrhoea in children can involve the administration of oral rehydration salts. These can replace bodily fluids and electrolytes that are wasted during diarrhoea, thus preventing dehydration. In the reviewed studies, children with diarrhoea were also administered zinc therapy. Zinc can epithelialise the intestinal wall, whose morphology and functioning are damaged during diarrhoea (1, 31). In three studies, oral rehydration salts were recommended (range = 5%–15%), and in three others, this treatment was combined with zinc therapy (range = 31.82%–58.3%). These findings differ from those derived in a study conducted in Surabaya, Indonesia, where only 2.38% of the pharmacists recommended oral rehydration salts and 2.38% suggested the oral rehydration salt-zinc combination (32). In a study in India, only 2.44% of the pharmacists recommended oral rehydration salts (13). In the Iraqi context, the pharmacists did not recommend antibiotic therapy, whether on its own or in combination, for children’s diarrhoea (26). This differs from research conducted in India, where 40.24% of the pharmacists recommended antibiotics as a therapy for paediatric diarrhoea (13). The current review uncovered that in three studies, the pharmacists suggested metronidazole as treatment (range = 18.18%–30%). Some cases of diarrhoea are caused by viruses, but the use of antibiotics will not shorten the duration of diarrhoea in children (3). In three studies, the pharmacists suggested loperamide (range = 4.54%–18%), and three others reported recommendations of anthelmintic therapy (range = 19.5%–22.72%). Loperamide and anthelmintics are not recommended as therapy for children with diarrhoea, with major side effects having been reported for the former (27).

For simulated patients to understand diarrhoea experienced by children, pharmacists should provide drug-related information to them. Pharmacists’ provision of such information can increase patient knowledge and compliance with therapy (12, 26). The results indicated that the pharmacists and pharmacy staff provided substantial drug-related information regarding their recommendations, namely, dosage (range = 19%–90.3%), drug administration frequency (range = 19%–72.6%), indications (range = 10%–80%) and adverse drug reactions (range = 13.3%–31.82%). Pharmacists can increase patient knowledge about diarrhoea therapy for children and accordingly deliver complete counselling. They can attend seminars or other continuing education programmes to increase their own knowledge (10).

Counselling by pharmacists regarding proper drug storage can prevent drug damage due to incorrect storage. If medicine is not stored in the right place, it can be reached and misused by children. Furthermore, many people keep medication at home without realising that they have expired (33). The review showed that only one study described how to store drugs properly (3.8%). Pharmacists can relay to patients that drugs should be stored in a medicine cabinet (34).

There are several limitations to a systematic literature review. The systematic design of the current work is its strength, but it also has certain shortcomings. This review identified eligible studies, but some may have been missed during the keyword search given the existence of multiple synonyms for ‘simulated patient’. Because this review was confined to studies published in English, those published in other languages were overlooked.

## Conclusion

The use of the patient simulation method in examining paediatric diarrhoea has increased in the pharmaceutical field over the last decade. There are differences in the manner by which patient history is taken, drug-related information is provided and therapeutic recommendations are provided in various countries. Pharmacies need to improve their services by elevating the quality of the aforementioned tasks in their establishments, which in turn, will increase the knowledge of simulated patients about treating diarrhoea in children.

## Figures and Tables

**Table 1:** Characteristics of individual studies and simulated patients

Authors	Setting	Study design	Instrument	Coverted/Consented	Scenarios	Simulated patient’s characteristics
Mesquita et al. (15)	The city of Aracaju in Brazil (25 pharmacies)	Cross-sectional	Scenario simulated patient’s, questionnaire, check list and audio-visual recorded (hidden micro camera)	Consented	A 3 years old child has diarrhoea for the past 2 days, with 2–3 liquid evacuations every day. She had a fever of 38.5 °C and vomited. Her brother goes to a pharmacy to get medication for his sister’s problems	Two undergraduate pharmacy students were trained as simulated patients. They visited community pharmacy once
Ogbo et al. (11)	The city of Lagos, Nigeria (186 pharmacies)	Cross-sectional	Scenario simulated patient’s, questionnaire and check list	Coverted	A 2.5 years old child has onset and duration of diarrhoea since yesterday with frequency of stooling since onset four times, no presence of fever and no presence blood and mucus in stool	Eight young mothers and caregivers were trained as simulated patients. They visited community pharmacy once
Sancar M et al. (16)	Two different districts of Istanbul, Turkey (100 community pharmacies)	Cross-sectional	Scenario simulated patient’s and check list	Consented	A 7-month-old baby who had experienced diarrhoea for 24 h (without any unusual smell or blood and with normal colour). The baby’s mother had stopped breast-feeding because of the diarrhoea. The frequency of faecal discharge was two-fold when compared with the patient’s normal pattern. Patients experienced mild vomiting; not presented fever or any sign of dehydration. Patient had no medication history and had never previously taken any medication for diarrhoea	One person was trained as simulated patients. They visited community pharmacy once
Abegaz et al. (17)	Community pharmacies from five towns of Northern Ethiopia	Cross-sectional	Scenario simulated patient’s, questionnaire and check list	Coverted	Under 5 years old children presence acute diarrhoea with no fever and no blood and mucus in stool	One person was trained as simulated patients. They visited community pharmacy once
Erku et al. (18)	Gondar town, Northwest Ethiopia (57 community medicine retail outlets: 20 community pharmacies, 35 drug stores and two rural drug vendors)	Cross-sectional	Scenario simulated patient’s, check list and audio records	Coverted	A 3-year-old child get serious diarrhoea. No presence of fever and no presence blood and mucus in stool	Four clinical pharmacists were trained as simulated patients. They visited community pharmacy once
Ayele et al. (19)	Gondar town, Ethiopia (Community drug retail outlets). Gondar town is located in Amhara regional state, Northwest Ethiopia	Cross-sectional	Scenario simulated patient’s, check list and audio records	Coverted	A 4-year-old child was suffering from acute diarrhoea. The diarrhoea starts yesterday afternoon (less than 1 day duration). There is no blood or mucus in the stool. No fever	Three graduating (fifth year) undergraduate pharmacy students were trained as simulated patients. They visited community pharmacy once
Mengistu et al. (20)	Dire Dawa city administration and Harar town, which are located in Eastern part of Ethiopia (105 community pharmacies)	Cross-sectional	Scenario simulated patient’s, questionnaire and check list	Coverted	A 3-year-old child with acute watery diarrhoea	A graduate of pharmacy was trained as simulated patients. They visited community pharmacy once
Malik et al. (21)	Lahore, Pakistan (Pharmacy and medical store)	Cross-sectional	Scenario simulated patient’s and check list	Coverted	A 3–5-year-old child having a problem of acute diarrhoea with no fever and no blood in the mucus	Eight undergraduate pharmacy students who have adequate medical background were trained as simulated patients. They visited community pharmacy once

**Table 2:** Counselling given by pharmacist and pharmacy staff

	Mesquita et al. (15)	Ogbo et al. (11)	Sancar et al. (16)	Abegaz et al. (17)	Erku et al. (18)	Ayele et al. (19)	Mengistu et al. (20)	Malik et al. (21)
History taken by pharmacist								
Age		23.1%	47%	90.3%			98.1%	
Frequency of stooling	12%	23.1%	8%	44.2%			24.8%	
Prescence mucus and blood in stool		23.1%	1%	49.6%			43.8%	
Pyrexia		23.1%	2%	41.6%			24.6%	
Symptoms	36%		9%					35.3%
Duration of patients diarrhoea/How long have you shown the signs/symptoms	36%		18%	67.3%			20%	
Vomiting			5%				19%	
Nutrition habits of patients			3%					
Medication history	12%		1%		16%	18.18%		32.5%
Allergy history				23.9%	6%	9.09%		4.4%
Patient condition	28%							57.2%
Chief complaint				23%				
Weight				23%				
Dispensed medication						95.45%		
Recommendation therapy by pharmacist								
Oral rehydration salt	8%	15%	5%					
Oral rehydration therapy + antibiotic, kaolin, metronidazole and antispasmodics		62.4%						
Antibiotic, kaolin, metronidazole and antispasmodics		22.5%						
No medication			8%					
Probiotics			18%					
Intestinal antiseptic			8%					
Commercial and handmade oral rehydration salt			3%					
Probiotics + intestinal antiseptic			5%					
Probiotic + antibiotic			1%					
Commercial and handmade oral rehydration salt + probiotics			1%					
Commercial and handmade oral rehydration salt + probiotics + intestinal antiseptic			1%					
Clarithromycin								0.13%
Erythromycin								0.26%
Amoxicillin								3.88%
Ciprofloxacin								6.21%
Cefixime								11.38%
Metronidazole					30%	18.18%		22.12%
Intestinal flora replenisher	92%							
Loperamide	8%				18%	4.54%		
Scopolamine	8%							
Dipyrone or dipyrone and associations	8%							
Oral rehydration salt + zinc				58.3%	32%	31.82%		
Antibacterial				51.3%				
Antiamoebic				20.4%				
Antihelmintic				19.5%	20%	22.72%		
Zinc				18.6%				
Antidiarrhoeal				15.9%				
Antispasmodic				13.3%				
Cotrimoxazole					22%	18.18%		
Drug information by pharmacist								
Instruction on food and fluid intake		7.5%						
Advise on fluid intake				50.4%				
Advise on food intake				30.9%				
Explain on how to use oral therapy salt sachet/oral rehydration salt preparation		26.4%					27.6%	
Aim of the medication			31%					
How to properly use medications			33%					
The duration of medication utilisation			23%					
Drug dispenses								50.7%
Patient reference to physician for better advice								2.9%
Indication	80%				10%	54.55%		
Pharmaceutical form	56%							
Dosage	36%			90.3%	19%	34.09%	90.5%	
Method for drug administration	38%						21.9%	
Drug administration times	40%			72.6%	19%	34.09%	61.9%	
Contraindications	4%							
Adherence to the treatment	4%							
Therapeutic alternative	28%							
Frequency				90.3%			82.9%	
Drug action				27.4%				
Adverse drug reaction				13.3%	26%	31.82%		
Non-pharmacological advice					12%	50%		
Name of medication							1.9%	
Storage conditions							3.8%	
Others								
Physician consultation made before coming to the pharmacy			2%					
Baby feeding			4%					

**Table 3:** Assessment using the criteria in the CRiSP checklist

No.	Checklist item	Mesquita et al. (15)	Ogbo et al. (11)	Sancar et al. (16)	Abegaz et al. (17)	Erku et al. (18)	Ayele et al. (19)	Mengistu et al. (20)	Malik et al. (21)
Title and background									
1.	Include the term simulated patient or some variant (e.g. mystery shopper).	Yes	Yes	Yes	Yes	Yes	Yes	Yes	Yes
2.	Describe the rationale, theory or goal behind using simulated patient methodology.	Yes	Yes	Yes	Yes	Yes	Yes	Yes	Yes
3.	Report the study design used (e.g. cross-sectional, case-control, randomised controlled trial, etc).	Yes	No	No	Yes	No	No	Yes	Yes
Simulated patients									
4a.	Report the number of simulated patients used in the study.	Yes	Yes	Yes	No	Yes	Yes	Yes	No
4b.	If more than one simulated patient was used, describe methods used to minimise variability between simulated patients.	Unclear	Unclear	Unclear	No	Unclear	Unclear	Unclear	Unclear
5.	Report demographics of the simulated patient(s) (include age, gender, qualifications (e.g. student, academic) and behaviour characteristics relevant to the scenario).	Yes	Yes	Yes	No	Yes	Yes	Yes	No
6.	Describe what was done during training sessions for simulated patients (e.g. role-play scenarios, etc.).	Yes	Yes	Unclear	No	Yes	Yes	Yes	No
7.	Actual: If simulated patient adherence or fidelity was assessed, describe the extent to which the scenario was delivered as planned.	Unclear	Unclear	Unclear	No	Unclear	Unclear	Unclear	Unclear
Simulated patient scenarios									
8a.	Describe how the scenarios were developed (including who they were developed by).	Yes	Yes	Yes	No	Yes	Yes	Yes	No
8b.	Describe if any guidelines (i.e. best practice guidelines) were used in development of the scenarios or if the scenarios were validated.	Yes	Yes	Yes	No	No	No	Yes	No
9a.	Give details about the scenario(s) used. Include any patient characteristics, patient prompts, scripts, props (e.g. prescriptions, medical devices), etc.	Yes	Yes	Yes	Yes	Yes	Yes	Yes	Yes
9b.	Describe any flexibility in scenarios or scripts to allow simulated patients to adapt based on participant responses.	Unclear	Unclear	Unclear	Unclear	Unclear	Unclear	Unclear	Unclear
10a.	Materials: Describe any physical or informational materials used in the intervention, including those provided to participants or used in intervention delivery or in training of intervention providers. Provide information on where the materials can be accessed (e.g. online appendix, URL).	Unclear	Unclear	Unclear	Unclear	Unclear	Unclear	Unclear	Unclear
10b.	Include a copy of any scripts or material given to simulated patients.	Unclear	Unclear	Unclear	Unclear	Unclear	Unclear	Unclear	Unclear
11.	Describe any intervention completed prior to the simulated patient assessment. Include procedures, activities, and/or processes (e.g. training sessions for health professionals).	Yes	Yes	Yes	No	Yes	Yes	Yes	No
12.	Procedures: Describe each of the procedures, activities, and/or processes used in the intervention, including any enabling or support activities.	Yes	Yes	Yes	No	Yes	Yes	Yes	No
13.	If the simulated patient assessment was modified (e.g. changes in personnel, assessment rubric, patient history or problems, etc.), describe these changes (what, why, when, and how).	No	No	No	No	No	No	No	No
14.	Describe any procedures that followed the simulated patient assessment (e.g. debriefs, performance feedback), including how (face to face, phone, etc.) and when these were conducted.	Yes	Yes	Yes	Yes	Yes	Yes	Yes	Yes
15.	Describe any procedures if simulated patients were identified by participants.	No	No	No	No	No	No	No	No
Data collection									
16.	Report how many simulated patient visits were conducted (include the planned number of visits, the number of actual completed visits, the number of visits per SP, number per scenario, number per health services provider e. g. pharmacy).	No	No	No	No	No	No	No	No
17.	Describe the mode(s) of delivery of the simulated patient assessment (e.g., face-to-face, telephone, internet, text, live, asynchronous, etc.).	Yes	Yes	Yes	Yes	Yes	Yes	Yes	Yes
18.	Describe the data collection procedure (e.g. data collection form, audio recording, telephone calls etc.).	Yes	Yes	Yes	No	Yes	Yes	Yes	No
19.	Describe how any data collection forms were created and validated (include a copy of any data collection forms if possible).	Yes	No	No	No	No	Yes	Yes	No
20	Describe when the data was collected by the simulated patient (i.e. during the visit, immediately after, etc.).	Yes	No	No	No	No	No	No	No
21	Address ways to avoid or minimise recall bias (e.g. the time taken to record data, use of audiotaping, use of an observer, etc).	Unclear	Unclear	Yes	Unclear	Yes	Yes	Unclear	Unclear
22	Report any conflicts of interest for assessors (e.g. if a simulated patient is a student assessing a colleague or preceptor).	Unclear	Unclear	Unclear	Unclear	Unclear	Unclear	Unclear	Unclear
Ethics									
23a.	Describe any ethics approval processes, consent from participants and ways of maintaining confidentiality.	Yes	Yes	Yes	Yes	Yes	Yes	No	Yes
23b.	Explain how participants were informed about being assessed using covert methods. If they were not, justify this.	Unclear	Unclear	Yes	Unclear	Unclear	Unclear	Unclear	Unclear
